# Systemic immune-inflammation index, thymidine phosphorylase and survival of localized gastric cancer patients after curative resection

**DOI:** 10.18632/oncotarget.9923

**Published:** 2016-06-08

**Authors:** Liu Huang, Shan Liu, Yu Lei, Kun Wang, Min Xu, Yaobing Chen, Bo Liu, Yangyang Chen, Qiang Fu, Peng Zhang, Kai Qin, Yixin Cai, Shengling Fu, Shuwang Ge, Xianglin Yuan

**Affiliations:** ^1^ Department of Oncology, Tongji Hospital, Tongji Medical College, Huazhong University of Science and Technology, Wuhan 430030, P.R. China; ^2^ The Second Clinical College, Tongji Medical College, Huazhong University of Science and Technology, Wuhan 430030, P.R. China; ^3^ Department of Pathology, Tongji Hospital, Tongji Medical College, Huazhong University of Science and Technology, Wuhan 430030, P.R. China; ^4^ Department of Thoracic Surgery, Tongji Hospital, Tongji Medical College, Huazhong University of Science and Technology, Wuhan 430030, P.R. China; ^5^ Department of Nephrology, Tongji Hospital, Tongji Medical College, Huazhong University of Science and Technology, Wuhan 430030, P.R. China

**Keywords:** gastric cancer, systemic immune-inflammation index, thymidine phosphorylase, peripheral blood counts, survival

## Abstract

Systemic immune-inflammation index (SII), based on lymphocyte (L), neutrophil (N), and platelet (P) counts, was recently developed and reflects comprehensively the balance of host inflammatory and immune status. We explored its prognostic value in localized gastric cancer (GC) after R0 resection and the potential associations with Thymidine phosphorylase (TYMP), which was reported to increase the migration and invasion of gastric cancer cells. A total of 455 GC patients who received D2 gastrectomy were enrolled. Blood samples were obtained within 1 week before surgery to measure SII (SII = P × N/L). TYMP expression was measured on tumor sections by immunohistochemical analysis. Preoperative high SII indicated worse prognosis (HR: 1.799; 95% CI: 1.174-2.757; *p* = 0.007) in multivariate analysis and was associated with higher pathological TNM stage, deeper local invasion of tumor and lymph node metastasis (all *p* < 0.001). SII predicted poor overall survival in pathological TNM stage I subgroup also (*p* < 0.001). Furthermore we found that in high SII group, positive rate of TYMP expression increased (53.7% vs 42.7%, *p* = 0.046) and TYMP positive patients had higher SII score (median 405.9 vs. 351.9, *p* = 0.026). SII, as a noninvasive and low cost prognostic marker, may be helpful to identify higher-risk patients after R0 resection, even for stage I GC patients.

## INTRODUCTION

Incidence rates of gastric cancer (GC) are highest in Eastern Asia (particularly in Korea, Mongolia, Japan, and China) [1]. Over the past couple of decades, advances have been achieved in surgery, chemotherapy, biological targeted therapy and radiotherapy. However, the prognosis of GC patients is still unsatisfactory. The most recent trends in 5-year Relative Survival Rates from 2005 to 2011 were less than 30% [2]. Patients with localized or regional GC were supposed to be curable. However, in spite of “curative” resection, approximate 35%-70% patients died within 5 years according to SEER database (http://seer.cancer.gov/statfacts/html/stomach.html). Therefore, it is of important significance to identify high risk subpopulations of recurrence or metastasis for tailoring rational adjuvant treatments to them after operation.

Recurrence and metastasis is the major cause of GC treatment failure and death. Previous studies showed that the circulating tumor cells (CTCs), inflammatory and immune cells such as platelets, neutrophils, and lymphocytes in the bloodstream played an important role in GC metastasis [3]. Platelets may act as protective “cloaks” for CTCs, shielding them from immune destruction, protect CTCs from shear stresses during circulation, induce epithelial-mesenchymal transition [4], and assist tumor cell to metastatic sites [5–7]. Neutrophils can promote the development and progression of cancer by providing an adequate tumor microenvironment via secretion of cytokines and chemokines [8]. Lymphocytes play a crucial role in cancer immune surveillance and defense by inducing cytotoxic cell death and inhibiting tumor cell proliferation and migration [9].

Recently, a novel systemic immune-inflammation index (SII) based on lymphocyte, neutrophil, and platelet counts was developed and has proved to be a powerful prognostic indicator of poor outcome for hepatocellular carcinoma and SCLC patients [10, 11]. However, the prognostic value of the preoperative SII in GC patients is still unclear. Thymidine phosphorylase (TYMP), also known as platelet-derived endothelial cell growth factor, has a role in tumorigenesis, angiogenesis, increasing cancer cell invasion activity and promoting cancer metastasis [12–14]. TYMP increases the migration and invasion of gastric cancer cells [13] and is associated with poorer survival rate [15, 16]. Blood platelets are one of the richest sources of TYMP. Was there any association between SII and TYMP? No relevant study has been done as far as we know. Here, we performed a large-scale retrospective cohort study to evaluate the prognostic value of SII in localized or regional GC patients after radical resection and investigate the associations of SII and TYMP expression.

## RESULTS

### Patient characteristics and their associations with overall survival (OS)

The clinicopathological characteristics and their associations with OS in 455 GC patients are summarized in Table 1. Stomach cancer rates are generally about twice as high in men as in women [1]. Similarly, in our cohort, 305 (67.0%) cases were males and 150 (33.0%) were females. Blood tests and TYMP expression in cancer tissues were not performed for 10 patients respectively; one patient neither had data of blood test nor TYMP expression. The median age was 56 years (range, 21-85). At the median follow-up of 655 days (range, 305-1017), 93 (20.4%) patients had died at last follow-up and the estimated mean OS was 831 days. Receiver operating characteristics (ROC) curve analysis determined the optimal cutoff value for SII was 571.28×10^9^ and we set it as 572×10^9^ to make it easier to be remembered (Table S1).

**Table 1:** The clinicopathological characteristics of patients and Kaplan-Meier analyses (log-rank test) of their predictive value on OS

	n	%	OS (days)				
			No. of events	mean survival	95% CI		*p*-value
**Age(y)**							0.234
≤ 50	131	28.8	32	752.885	694.26	811.51	
> 50	324	71.2	61	844.22	806.634	881.805	
Median (range)	56(21-85)						
**Sex**							0.625
male	305	67.0	60	837.261	797.832	876.691	
female	150	33.0	33	772.708	720.629	824.787	
**Tumor differentiation**							<0.001 (9.45E-05)
G1	38	8.4	1	885.500	831.414	939.586	
G2	193	42.4	35	851.104	802.927	899.28	
G3	186	40.9	45	753.595	705.069	802.12	
Mucinous adenocarcinoma	17	3.7	1	866.235	764.003	968.468	
Signet ring cell carcinoma	21	4.6	11	565.178	410.578	719.778	
**Lauren classification**							0.289
intestinal type	335	73.6	66	837.305	799.707	874.903	
diffuse type	38	8.4	12	703.724	593.202	814.246	
mixed type	82	18.0	15	778.464	714.768	842.159	
**Tumor site**							0.196
Upper one-third/Cardia	69	15.2	19	772.059	685.282	858.836	
Middle one-third	178	39.1	30	864.453	816.441	912.465	
lower one-third	208	45.7	44	770.669	725.636	815.701	
**Pathological TNM stage**							<0.001 (5.99E-11)
StageI (IA/IB)	120	26.4	4	920.141	895.06	945.221	
StageII (IIA/IIB)	108	23.7	15	883.541	823.328	943.754	
StageIII (IIIA/IIIB/IIIC)	227	49.9	74	675.781	628.214	723.347	
**T stage**							<0.001 (8.22E-07)
T1	96	21.1	3	920.821	892.625	949.018	
T2	59	13.0	8	806.526	738.635	874.417	
T3	50	11.0	9	797.332	711.579	883.086	
T4	250	54.9	73	754.549	705.547	803.551	
**N stage**							<0.001 (7.27E-12)
N0	188	41.3	15	941.968	908.067	975.869	
N1	66	14.5	9	835.202	769.567	900.837	
N2	79	17.4	21	689.154	611.773	766.534	
N3	122	26.8	48	618.658	552.33	684.985	
**SII**							<0.001 (5.01E-05)
<572	335	75.3	55	868.166	833.559	902.773	
≥572	110	24.7	36	635.978	571.650	700.306	

OS, Overall survival; SII, systemic immune-inflammation index; CI, confidence interval.

The univariate cox analysis indicated that the significant prognostic factors were tumor differentiation, T stage, N stage, pathological tumor-nodes-metastasis (TNM) stage and SII, all with a *p*-value < 0.001 (Table 1). Neither platelet-lymphocyte ratio (PLR) nor neutrophil-lymphocyte ratio (NLR) was associated with OS (Table S2).

**Table 2:** Multivariate cox regression analyses of OS (enter, n = 445)

	HR	95% CI		*p*-value
Pathological TNM stage	2.685	1.843-	3.910	<0.001 (2.63E-07)
Tumor differentiation	1.413	1.128-	1.770	0.003
SII	1.799	1.174-	2.757	0.007

OS, Overall survival; SII, systemic immune-inflammation index; CI, confidence interval; HR, Hazard ratios.

### Multivariate cox proportional hazard analysis for OS

**Figure 1:**
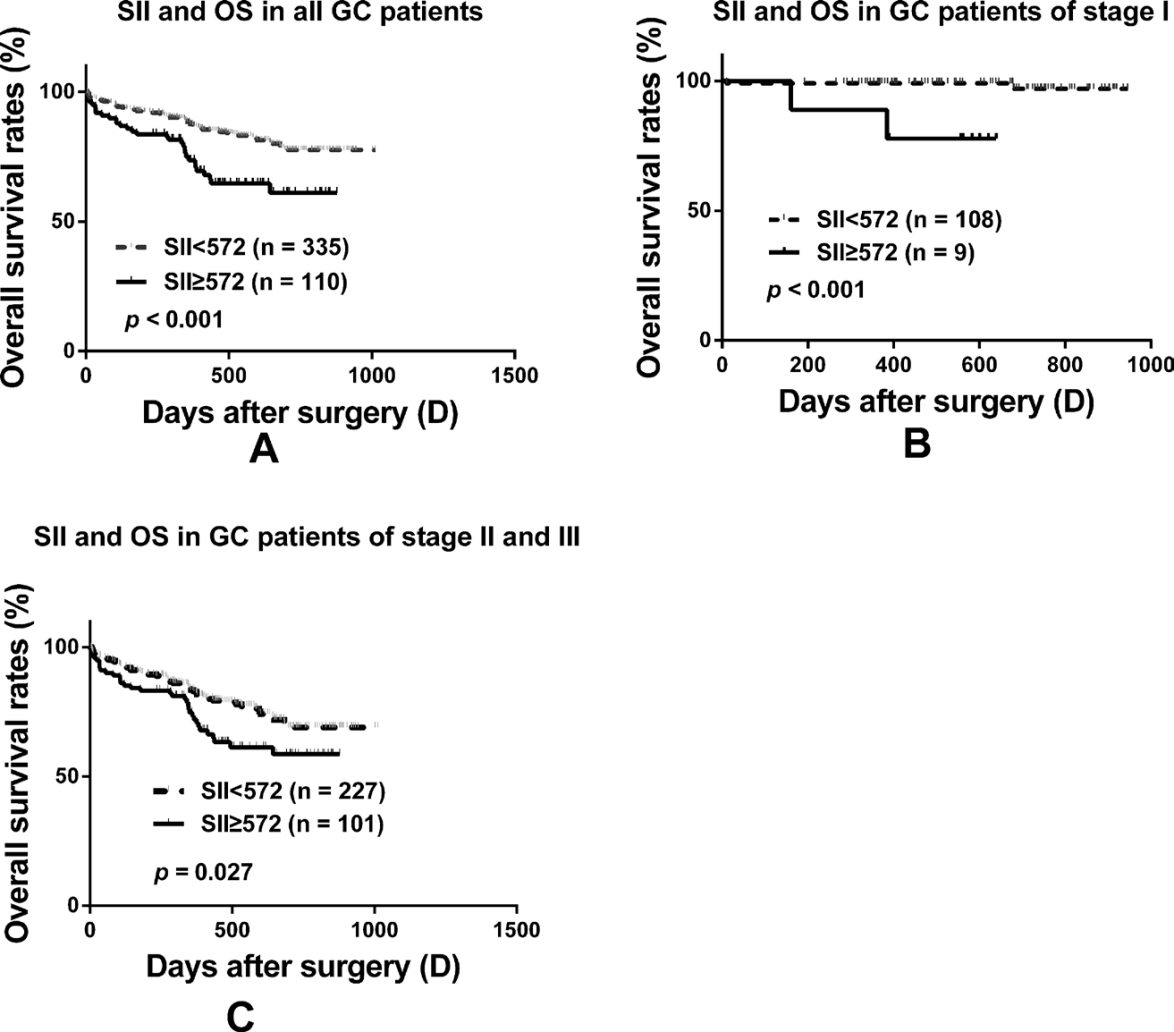
Prognostic significance of SII in GC patients undergoing R0 resection Kaplan-Meier analysis of OS for the SII in all GC patients after curative resection **A.**, in stage I **B.** and in stage II+III **C.** respectively.

In the multivariate analysis, SII (HR: 1.799; 95% CI: 1.174-2.757; *p* = 0.007), together with pathological TNM stage (HR: 2.685; 95% CI: 1.843-3.910; *p* = 2.63E-07) and tumor differentiation (HR: 1.413; 95% CI: 1.128-1.770; *p* = 0.003) were identified to be the independent prognostic factors, after adjustment for other characteristics (Table 2). SII was significantly associated with OS in GC patients after D2 resection and the survival curves are presented in Figure 1A, *p* <0.001.

### Association of the SII with clinicopathological parameters

We found that patients with an SII ≥ 572 were more likely to have higher pathological TNM stage (*p* = 2.10E-06), deeper local invasion of tumor (*p* = 2.66E-05) and lymph node metastasis (*p* = 3.72E-05). Results are shown in Table 3.

**Table 3:** Correlations between SII and clinicopathological characteristics (n=445)

Variables		SII		*p*-value
		<572	≥572	
**No. of patients**		335	110	
**Age (y)**	≤50	94	34	0.567
	>50	241	76	
**Sex**	Male	217	79	0.175
	Female	118	31	
**Pathological TNM stage**	StageI (IA and IB)	108	9	**2.10E-06**
	StageII (IIA and IIB)	77	28	
	StageIII (IIIA and IIIB)	150	73	
**T stage**	T1	87	7	**2.66E-05**
	T2	46	10	
	T3	33	16	
	T4a	169	77	
**N stage**	N0	157	27	**2.15E-04**
	N1	47	18	
	N2	56	22	
	N3	75	43	
**Lymph node metastasis**	no	157	27	**3.72E-05**
	yes	178	83	
**Tumor differentiation**	G1	33	4	0.227
	G2	138	51	
	G3	135	48	
	Mucinous adenocarcinoma	13	2	
	Signet ring cell carcinoma	16	5	
TYMP expression^*^	negative	188	50	**0.046**
	positive	140	58	

SII, systemic immune-inflammation index; TYMP, thymidine phosphorylase;

* TYMP expression and SII was avalible in 436 patients.

### The prognostic significance of SII in patients with different TNM stage

We investigated the prognostic significance of the SII in GC patients with different TNM stage in greater detail. We found that the SII was significantly correlated with OS in different pathological TNM stage subgroup (Figure 1B and Figure 1C), even in the group of stage I (Figure 1B, *p* <0.001).

### Correlation between the SII and TYMP expression

**Figure 2:**
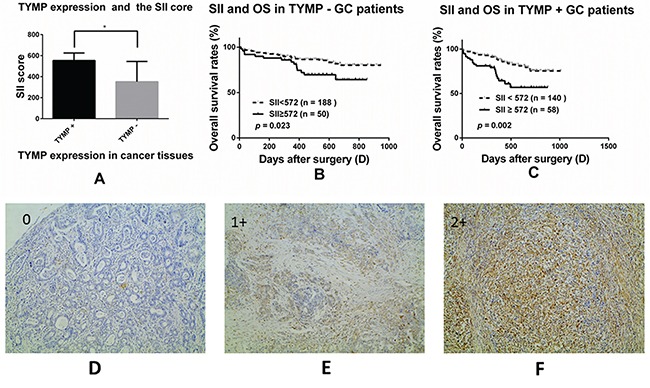
The prognostic significance of the SII in GC patients with different thymidine phosphorylase (TYMP) expression subgroups The correlation between the SII and TYMP expression **A.**, *, *p* = 0.026; Kaplan-Meier analysis of OS for the SII in TYMP negative **B.** and TYMP positive patients **C.**; Immunohistochemical staining for TYMP in cancer tissues (original magnification × 100). TYMP = 0, less than 10% of the cancer cells were TYMP-positive **D.**; TP = 1+, more than 10% but less than 50% of the cancer cells were TYMP-positive **E.**; TP = 2+, more than 50% of the cancer cells were TYMP-positive **F.**. TYMP = 0 was considered as TYMP negative while TYMP = 1+ or 2+ was considered as TYMP positive.

The correlation between preoperative SII score and TYMP expression was further investigated. GC patients with positive TYMP expression had higher SII score, the median SII was 405.9 vs. 351.9, *p* = 0.026 (Figure 2A). And, the TYMP positive rate was 42.7% (140/328) in the group of SII < 572 and 53.7% (58/108) in the group of SII ≥ 572 respectively, *p* = 0.046 (Table 3).

### The prognostic significance of SII in GC patients with different TYMP expression

We divided all GC patients into two groups according to the expression status of TYMP in cancer tissues. The SII score significantly correlated with OS in both TYMP positive group (Figure 2C, *p* = 0.002) and TYMP negative group (Figure 2B, *p* = 0.023). Patients of high SII and with positive TYMP expression may have the worst survival (Figure S1, *p* = 0.040).

## DISCUSSION

Several studies have analyzed the prognostic significance of peripheral blood values, such as neutrophil-lymphocyte ratio (NLR), platelet-lymphocyte ratio (PLR) and platelet counts in patients with localized or regional GC or colorectal cancer. However, the results were inconsistent [17–26]. A novel immune-inflammation based prognostic score (SII), based on lymphocyte, neutrophil, and platelet counts, was shown to be an independent predictor of recurrence and survival for HCC patients [10, 27] and for SCLC patients [11]. SII, as a prognostic factor for poor survival, was considered to be superior to NLR and PLR, and was related to higher CTCs levels [10]. In our study, we confirmed the prognostic value of preoperative SII, as a noninvasive, low cost, easily assessable and reproducible prognostic parameter, in GC patients after R0 resection. We found that high preoperative SII indicated a worse prognosis and was associated with poor clinicopathological prognostic factors.

Several studies may give several explanations for our results: (1) Cytotoxic lymphocytes and other lymphocytes play a fundamental role in cell-mediated immunologic destruction of cancer cells [28]. Meanwhile, they can release several cytokines, such as IFN-γ and TNF-α, to promote tumor control and improve prognosis of cancer patients. (2) Neutrophils have a crucial role in the pathogenesis of a broad range of diseases, including cancer [29]. It can enhance the invasion, proliferation, and metastasis of cancer cells as well as aid them to evade immune surveillance [9]. (3) Platelet-derived signals recruit granulocytes and guide the formation of early metastatic niches, which are crucial for metastasis [30]. Tumor-associated platelets release ATP into the blood and facilitate tumor metastasis by relaxing endothelial barrier function [5]. Platelets have direct contact with tumor cells, synergistically activate the TGFbeta/Smad and NF-kappaB pathways in cancer cells, induce an epithelial-mesenchymal-like transition and promote metastasis [4]. Thus, an elevated SII, due to high levels of neutrophils and platelets while low level of lymphocytes, usually suggests a stronger inflammatory and a weaker immune response in patients. It may be associated with invasion and metastasis of cancer cells and hence lead to poor survival.

In the subgroup analysis, we furthermore found that, the prognostic significance of SII was duplicated in localized GC patients with different TYMP expression of cancer tissues and American Joint Committee on Cancer (AJCC) TNM classification. SII can even predict OS of stage I GC patients.

TYMP was proved to be an angiogenesis-promoting factor [31]. It plays an important role in angiogenesis and extracellular matrix remodeling and can thus stimulate tumor growth and metastasis [12]. High level of TYMP in cancer patients resulted in more aggressive cancer growth, higher incidence of vascular infiltration and metastasis and may thereby lead to unfavorable survival [12, 15, 16, 32]. We found that SII was positively associated with TYMP expression. It may partly explain why SII can influence cancer progression and survival.

According to our research, SII can predict poor survival of GC patients with stage I. It was meaningful because GC patients have poor outcomes. In spite of “curative” resection, 35%-70% localized GC patients died of recurrence and metastasis within 5 years. So far there were no suitable markers to predict recurrence or metastasis in early stage GC patients, SII, as a noninvasive, low cost, easy and reproducible prognostic marker, may be helpful to further identify higher-risk GC patients after R0 resection.

Suee Lee etc. previously reported that the normalization of neutrophil lymphocyte ratio and platelet lymphocyte ratio after FOLFOX chemotherapy was associated with longer OS in GC patients [33], thereby demonstrating that change of neutrophils, platelets and lymphocytes during anti-cancer therapy may also affect treatment outcomes. Granulocyte or macrophage colony stimulating factor (G/M-CSF) is now widely used during anti-cancer treatment processes and some of patients have even received them preventively. Our results may alarm us to avoid the overuse of G/M-CSF.

In conclusion, preoperative SII can predict OS of localized GC patients after R0 resection, even for those who are of stage I. For patients with higher SII (> 572) before surgery, it is more advisable to pay attention when making adjuvant treatment plans.

## MATERIALS AND METHODS

### Patient eligibility and study design

This study was approved by the Ethics Committee of Huazhong University of Science and Technology. From January 2013 to December 2014, a total of 455 consecutive GC patients who received D2 gastrectomy with R0 resection at Tongji hospital were enrolled. The seventh edition of the AJCC TNM staging classification for carcinoma of the stomach was used for tumor staging. Other eligibility criteria included histologically confirmed adenocarcinoma of the stomach; stage I to III depending on postoperative histological specimen. Potential prognostic factors were gathered, including age, sex, preoperative laboratory measurements, postoperative tumor characteristics etc.

Blood samples were obtained within 1 week before surgery to measure the neutrophil, lymphocyte, and platelet counts. Exclusion criteria included active infection or inflammatory disease within 1 month before blood test; metastasis accrued within 3 months after surgery; patients who received neoadjuvant chemotherapy or radiotherapy. Patients were followed up carefully after surgery at 6- to 12-month intervals. The last follow-up date was October 17, 2015. OS was calculated from the date of surgery to the date of death or last follow-up. SII = P × N/L, where P, N, and L were the preoperative absolute platelet, neutrophil, and lymphocyte counts, respectively [10]. Tumor differentiation was classified as G1, G2, G3 adenocarcinoma and signet ring cell carcinoma. Lauren classification was defined as intestinal type, diffuse type and mixed type.

### Expression of TYMP in cancer tissues

Immunohistochemical analysis of TYMP was performed on formalin fixed, paraffin embedded tissue sections (4 μm) using a labeled streptavidin biotin method (LSAB kit; Zhongshanjinqiao Beijin). The concentration of the mouse anti-TYMP antibody was 100μg/mL; working dilution was 1:50. The second antibody was biotinylated rabbit anti-rat immunoglobulin (Zhongshanjinqiao Beijing). Tumor sections stained for TYMP were scored based on percentage of positive cells. The scale was 0: positive cells < 10% (Figure 2D), 1: positive cells 10%-50% (Figure 2E), 2: positive cells >50% (Figure 2F). Two researchers who had no knowledge of the patient's clinicopathological factors evaluated the expression score. A third pathologist resolved the inconsistencies.

### Statistical analysis

The univariate cox regression analyses were performed to evaluate the associations between SII, platelet-lymphocyte ratio (PLR), neutrophil-lymphocyte ratio (NLR), clinicopathological factors and OS. Only those variables that proved to be significant in the univariate analysis were included in the final multivariate cox regression model (enter). HR and their 95% CI were calculated as estimates of the correlations. Kaplan-Meier analysis and log-rank test were performed to estimate the distribution of OS and to compare the differences between survival curves. ROC analysis and the Youden index were performed to identify the optimal cutoff value of SII for OS (Table S1). Subsequently, the SII was stratified into < 572×10^9^ or ≥ 572×10^9^ for subsequent analysis. The Pearson Chi-square test or Fisher's exact test were used to compare qualitative variables. Student's t test or Mann Whitney test were performed to compare quantitative variables depending on the result of homogeneity of variance test, such as to compare SII level in patients with or without TYMP expression. A value of *p* < 0.05 was considered statistically significant in a two-tailed test. Statistical analysis was performed using SPSS 16.0 statistical software (SPSS Inc., Chicago, IL) and Graphpad Prism 6.

## SUPPLEMENTARY FIGURE AND TABLES




