# Supplementary material for: Systemic immune-inflammation index, thymidine phosphorylase and survival of localized gastric cancer patients after curative resection
Source: Oncotarget. 2016 Jun 8;7(28):44185–93. doi: 10.18632/oncotarget.9923 (PMC5190088; doi:10.18632/oncotarget.9923)
Supplement: Supplementary file 1 [file oncotarget-07-44185-s001.pdf]

Systemic immune-inflammation index, thymidine phosphorylase and survival of localized gastric cancer patients after curative resection

SUPPLEMENTARY FIGURE AND TABLES

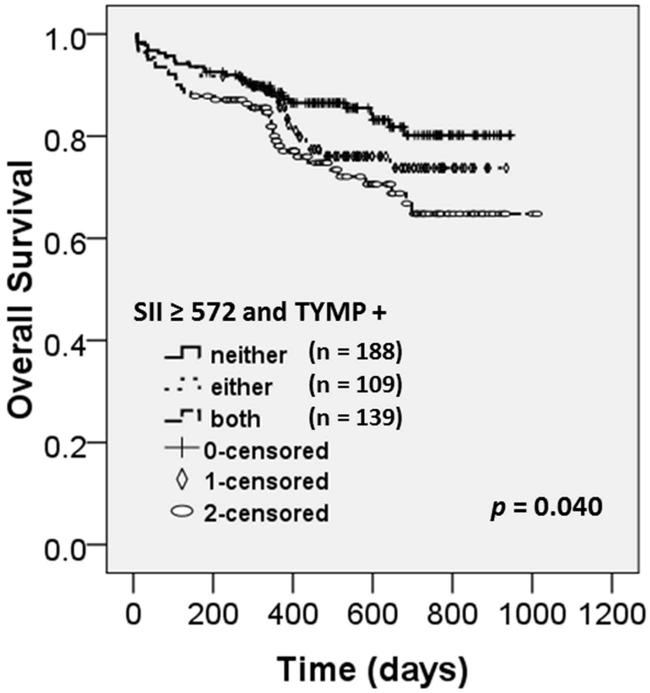

Supplementary Figure S1: The prognostic significance of SII and thymidine phosphorylase (TYMP) expression in gastric cancer (GC) patients. 0 = SII < 572 / TYMP negative; 1 = SII ≥ 572 / TYMP negative or SII < 572 / TYMP positive, ; 2 = SII ≥ 572 / TYMP positive.

Supplementary Table S1: Receiver operating characteristics (ROC) analysis for the cut-off value of SII

See Supplementary File 1

Supplementary Table S2: The univariate Cox analyses of OS (n = 455)

	HR	95% CI		*p*-value
Age(y)	0.772	0.503	1.184	0.236
sex	1.112	0.727	1.700	0.625
Tumor differentiation	1.459	1.188	1.793	< 0.001
Lauren classification	1.002	0.773	1.298	0.990
Tumor site	0.967	0.730	1.283	0.818
Pathological TNM stage	3.134	2.157	4.555	< 0.001
T stage	1.887	1.475	2.413	< 0.001
N stage	1.854	1.549	2.218	< 0.001
NLR*	0.996	0.956	1.038	0.862
PLR*	0.999	0.998	1.000	0.268
SII*	1.001	1.000	1.001	< 0.001

*, n = 445; NLR, neutrophil-lymphocyte ratio; PLR, platelet-lymphocyte ratio; SII, systemic immune-inflammation index; CI, confidence interval.
