# Supplementary material for: Systemic immune-inflammation index, thymidine phosphorylase and survival of localized gastric cancer patients after curative resection
Source: Oncotarget. 2016 Jun 8;7(28):44185–93. doi: 10.18632/oncotarget.9923 (PMC5190088; doi:10.18632/oncotarget.9923)
Supplement: Supplementary file 2 [file oncotarget-07-44185-s002.docx]

Table S1. Receiver operating characteristics (ROC) analysis for the cut-off value of SII

| Positive if Greater Than or Equal Toa | Sensitivity | 1 - Specificity | Youden index |
| --- | --- | --- | --- |
| 244.61264 | 0.77778 | 0.77841 | -0.00063 |
| 74.87097 | 1.00000 | 1.00000 | 0.00000 |
| 4231.00000 | 0.00000 | 0.00000 | 0.00000 |
| 245.65482 | 0.77778 | 0.77557 | 0.00221 |
| 76.22336 | 1.00000 | 0.99716 | 0.00284 |
| 246.17442 | 0.77778 | 0.77273 | 0.00505 |
| 242.92478 | 0.78889 | 0.78125 | 0.00764 |
| 246.75266 | 0.77778 | 0.76989 | 0.00789 |
| 253.80549 | 0.76667 | 0.75852 | 0.00814 |
| 85.08369 | 1.00000 | 0.99148 | 0.00852 |
| 243.58772 | 0.78889 | 0.77841 | 0.01048 |
| 247.90774 | 0.77778 | 0.76705 | 0.01073 |
| 254.07264 | 0.76667 | 0.75568 | 0.01098 |
| 4003.69149 | 0.01111 | 0.00000 | 0.01111 |
| 99.56504 | 1.00000 | 0.98864 | 0.01136 |
| 248.65329 | 0.77778 | 0.76420 | 0.01357 |
| 2595.78065 | 0.02222 | 0.00852 | 0.01370 |
| 254.41752 | 0.76667 | 0.75284 | 0.01383 |
| 106.27220 | 1.00000 | 0.98580 | 0.01420 |
| 249.76434 | 0.77778 | 0.76136 | 0.01641 |
| 2858.16725 | 0.02222 | 0.00568 | 0.01654 |
| 256.54614 | 0.76667 | 0.75000 | 0.01667 |
| 107.88328 | 1.00000 | 0.98295 | 0.01705 |
| 242.38651 | 0.80000 | 0.78125 | 0.01875 |
| 252.22260 | 0.77778 | 0.75852 | 0.01926 |
| 3225.64939 | 0.02222 | 0.00284 | 0.01938 |
| 259.14192 | 0.76667 | 0.74716 | 0.01951 |
| 110.85729 | 1.00000 | 0.98011 | 0.01989 |
| 238.95343 | 0.81111 | 0.78977 | 0.02134 |
| 3554.31649 | 0.02222 | 0.00000 | 0.02222 |
| 261.28227 | 0.76667 | 0.74432 | 0.02235 |
| 114.58834 | 1.00000 | 0.97727 | 0.02273 |
| 240.37870 | 0.81111 | 0.78693 | 0.02418 |
| 2556.55544 | 0.03333 | 0.00852 | 0.02481 |
| 263.02848 | 0.76667 | 0.74148 | 0.02519 |
| 117.11137 | 1.00000 | 0.97443 | 0.02557 |
| 241.19311 | 0.81111 | 0.78409 | 0.02702 |
| 263.49666 | 0.76667 | 0.73864 | 0.02803 |
| 201.72621 | 0.88889 | 0.86080 | 0.02809 |
| 120.32231 | 1.00000 | 0.97159 | 0.02841 |
| 241.93473 | 0.81111 | 0.78125 | 0.02986 |
| 263.77858 | 0.76667 | 0.73580 | 0.03087 |
| 202.74249 | 0.88889 | 0.85795 | 0.03093 |
| 124.57735 | 1.00000 | 0.96875 | 0.03125 |
| 140.57506 | 0.98889 | 0.95739 | 0.03150 |
| 237.41048 | 0.82222 | 0.78977 | 0.03245 |
| 264.11250 | 0.76667 | 0.73295 | 0.03371 |
| 205.71556 | 0.88889 | 0.85511 | 0.03378 |
| 128.10471 | 1.00000 | 0.96591 | 0.03409 |
| 145.16092 | 0.98889 | 0.95455 | 0.03434 |
| 293.85705 | 0.70000 | 0.66477 | 0.03523 |
| 2412.67705 | 0.04444 | 0.00852 | 0.03592 |
| 265.90272 | 0.76667 | 0.73011 | 0.03655 |
| 207.97400 | 0.88889 | 0.85227 | 0.03662 |
| 131.60929 | 1.00000 | 0.96307 | 0.03693 |
| 147.06960 | 0.98889 | 0.95170 | 0.03718 |
| 294.00666 | 0.70000 | 0.66193 | 0.03807 |
| 201.39497 | 0.90000 | 0.86080 | 0.03920 |
| 267.74442 | 0.76667 | 0.72727 | 0.03939 |
| 209.18942 | 0.88889 | 0.84943 | 0.03946 |
| 134.12483 | 1.00000 | 0.96023 | 0.03977 |
| 147.39352 | 0.98889 | 0.94886 | 0.04003 |
| 236.75532 | 0.83333 | 0.79261 | 0.04072 |
| 294.20566 | 0.70000 | 0.65909 | 0.04091 |
| 268.87190 | 0.76667 | 0.72443 | 0.04223 |
| 211.19351 | 0.88889 | 0.84659 | 0.04230 |
| 136.27361 | 1.00000 | 0.95739 | 0.04261 |
| 148.00201 | 0.98889 | 0.94602 | 0.04287 |
| 231.80981 | 0.84444 | 0.80114 | 0.04331 |
| 236.97726 | 0.83333 | 0.78977 | 0.04356 |
| 294.45962 | 0.70000 | 0.65625 | 0.04375 |
| 197.33366 | 0.91111 | 0.86648 | 0.04463 |
| 269.88404 | 0.76667 | 0.72159 | 0.04508 |
| 212.78083 | 0.88889 | 0.84375 | 0.04514 |
| 275.81668 | 0.75556 | 0.71023 | 0.04533 |
| 149.21858 | 0.98889 | 0.94318 | 0.04571 |
| 290.29130 | 0.72222 | 0.67614 | 0.04609 |
| 233.05302 | 0.84444 | 0.79830 | 0.04615 |
| 293.71209 | 0.71111 | 0.66477 | 0.04634 |
| 295.23952 | 0.70000 | 0.65341 | 0.04659 |
| 2291.33965 | 0.05556 | 0.00852 | 0.04703 |
| 199.03303 | 0.91111 | 0.86364 | 0.04747 |
| 270.33313 | 0.76667 | 0.71875 | 0.04792 |
| 213.65208 | 0.88889 | 0.84091 | 0.04798 |
| 276.94645 | 0.75556 | 0.70739 | 0.04817 |
| 150.83385 | 0.98889 | 0.94034 | 0.04855 |
| 228.60839 | 0.85556 | 0.80682 | 0.04874 |
| 290.40793 | 0.72222 | 0.67330 | 0.04893 |
| 234.76890 | 0.84444 | 0.79545 | 0.04899 |
| 296.39170 | 0.70000 | 0.65057 | 0.04943 |
| 200.52426 | 0.91111 | 0.86080 | 0.05032 |
| 270.99536 | 0.76667 | 0.71591 | 0.05076 |
| 214.83647 | 0.88889 | 0.83807 | 0.05082 |
| 277.99030 | 0.75556 | 0.70455 | 0.05101 |
| 226.10444 | 0.86667 | 0.81534 | 0.05133 |
| 152.56379 | 0.98889 | 0.93750 | 0.05139 |
| 229.63530 | 0.85556 | 0.80398 | 0.05158 |
| 290.89286 | 0.72222 | 0.67045 | 0.05177 |
| 236.07741 | 0.84444 | 0.79261 | 0.05183 |
| 297.22306 | 0.70000 | 0.64773 | 0.05227 |
| 271.88415 | 0.76667 | 0.71307 | 0.05360 |
| 215.71351 | 0.88889 | 0.83523 | 0.05366 |
| 279.40951 | 0.75556 | 0.70170 | 0.05385 |
| 222.39310 | 0.87778 | 0.82386 | 0.05391 |
| 227.68632 | 0.86667 | 0.81250 | 0.05417 |
| 154.88471 | 0.98889 | 0.93466 | 0.05423 |
| 231.07662 | 0.85556 | 0.80114 | 0.05442 |
| 291.85600 | 0.72222 | 0.66761 | 0.05461 |
| 184.43070 | 0.95556 | 0.90057 | 0.05499 |
| 297.54990 | 0.70000 | 0.64489 | 0.05511 |
| 196.05580 | 0.92222 | 0.86648 | 0.05574 |
| 273.69561 | 0.76667 | 0.71023 | 0.05644 |
| 215.98016 | 0.88889 | 0.83239 | 0.05650 |
| 280.87714 | 0.75556 | 0.69886 | 0.05669 |
| 224.16778 | 0.87778 | 0.82102 | 0.05676 |
| 228.07223 | 0.86667 | 0.80966 | 0.05701 |
| 156.56727 | 0.98889 | 0.93182 | 0.05707 |
| 290.14455 | 0.73333 | 0.67614 | 0.05720 |
| 292.98849 | 0.72222 | 0.66477 | 0.05745 |
| 185.13450 | 0.95556 | 0.89773 | 0.05783 |
| 297.98994 | 0.70000 | 0.64205 | 0.05795 |
| 2221.69855 | 0.06667 | 0.00852 | 0.05814 |
| 217.85422 | 0.88889 | 0.82955 | 0.05934 |
| 282.27237 | 0.75556 | 0.69602 | 0.05953 |
| 224.37893 | 0.87778 | 0.81818 | 0.05960 |
| 288.15645 | 0.74444 | 0.68466 | 0.05979 |
| 228.31247 | 0.86667 | 0.80682 | 0.05985 |
| 157.63253 | 0.98889 | 0.92898 | 0.05991 |
| 186.37887 | 0.95556 | 0.89489 | 0.06067 |
| 299.07403 | 0.70000 | 0.63920 | 0.06080 |
| 219.63298 | 0.88889 | 0.82670 | 0.06218 |
| 284.53316 | 0.75556 | 0.69318 | 0.06237 |
| 224.70347 | 0.87778 | 0.81534 | 0.06244 |
| 289.08523 | 0.74444 | 0.68182 | 0.06263 |
| 158.69955 | 0.98889 | 0.92614 | 0.06275 |
| 187.37245 | 0.95556 | 0.89205 | 0.06351 |
| 302.22826 | 0.70000 | 0.63636 | 0.06364 |
| 189.43886 | 0.94444 | 0.88068 | 0.06376 |
| 220.23921 | 0.88889 | 0.82386 | 0.06503 |
| 286.40848 | 0.75556 | 0.69034 | 0.06521 |
| 289.31549 | 0.74444 | 0.67898 | 0.06547 |
| 159.53568 | 0.98889 | 0.92330 | 0.06559 |
| 182.29086 | 0.96667 | 0.90057 | 0.06610 |
| 187.38269 | 0.95556 | 0.88920 | 0.06635 |
| 304.86961 | 0.70000 | 0.63352 | 0.06648 |
| 190.51536 | 0.94444 | 0.87784 | 0.06660 |
| 195.22965 | 0.93333 | 0.86648 | 0.06686 |
| 286.90476 | 0.75556 | 0.68750 | 0.06806 |
| 289.71659 | 0.74444 | 0.67614 | 0.06831 |
| 160.24361 | 0.98889 | 0.92045 | 0.06843 |
| 187.49160 | 0.95556 | 0.88636 | 0.06919 |
| 1878.68352 | 0.07778 | 0.00852 | 0.06926 |
| 305.68035 | 0.70000 | 0.63068 | 0.06932 |
| 192.20251 | 0.94444 | 0.87500 | 0.06944 |
| 790.01540 | 0.18889 | 0.11932 | 0.06957 |
| 287.29264 | 0.75556 | 0.68466 | 0.07090 |
| 160.87000 | 0.98889 | 0.91761 | 0.07128 |
| 1381.92860 | 0.10000 | 0.02841 | 0.07159 |
| 188.07102 | 0.95556 | 0.88352 | 0.07203 |
| 306.44822 | 0.70000 | 0.62784 | 0.07216 |
| 193.64449 | 0.94444 | 0.87216 | 0.07229 |
| 796.30090 | 0.18889 | 0.11648 | 0.07241 |
| 161.50833 | 0.98889 | 0.91477 | 0.07412 |
| 1387.30775 | 0.10000 | 0.02557 | 0.07443 |
| 188.63103 | 0.95556 | 0.88068 | 0.07487 |
| 307.47355 | 0.70000 | 0.62500 | 0.07500 |
| 194.12498 | 0.94444 | 0.86932 | 0.07513 |
| 797.51637 | 0.18889 | 0.11364 | 0.07525 |
| 921.87657 | 0.15556 | 0.07955 | 0.07601 |
| 162.46056 | 0.98889 | 0.91193 | 0.07696 |
| 179.07105 | 0.97778 | 0.90057 | 0.07721 |
| 1420.69989 | 0.10000 | 0.02273 | 0.07727 |
| 309.65537 | 0.70000 | 0.62216 | 0.07784 |
| 194.69463 | 0.94444 | 0.86648 | 0.07797 |
| 799.64066 | 0.18889 | 0.11080 | 0.07809 |
| 833.05374 | 0.17778 | 0.09943 | 0.07835 |
| 901.67713 | 0.16667 | 0.08807 | 0.07860 |
| 931.30921 | 0.15556 | 0.07670 | 0.07885 |
| 1122.72527 | 0.13333 | 0.05398 | 0.07936 |
| 164.23559 | 0.98889 | 0.90909 | 0.07980 |
| 1276.02632 | 0.11111 | 0.03125 | 0.07986 |
| 1468.35239 | 0.10000 | 0.01989 | 0.08011 |
| 1561.10065 | 0.08889 | 0.00852 | 0.08037 |
| 311.47794 | 0.70000 | 0.61932 | 0.08068 |
| 783.87199 | 0.20000 | 0.11932 | 0.08068 |
| 802.97810 | 0.18889 | 0.10795 | 0.08093 |
| 843.31024 | 0.17778 | 0.09659 | 0.08119 |
| 912.28716 | 0.16667 | 0.08523 | 0.08144 |
| 946.88175 | 0.15556 | 0.07386 | 0.08169 |
| 404.16095 | 0.52222 | 0.44034 | 0.08188 |
| 1132.59570 | 0.13333 | 0.05114 | 0.08220 |
| 166.61316 | 0.98889 | 0.90625 | 0.08264 |
| 1328.97225 | 0.11111 | 0.02841 | 0.08270 |
| 1488.74074 | 0.10000 | 0.01705 | 0.08295 |
| 312.93604 | 0.70000 | 0.61648 | 0.08352 |
| 806.47873 | 0.18889 | 0.10511 | 0.08378 |
| 860.21541 | 0.17778 | 0.09375 | 0.08403 |
| 914.59239 | 0.16667 | 0.08239 | 0.08428 |
| 962.50994 | 0.15556 | 0.07102 | 0.08453 |
| 405.03649 | 0.52222 | 0.43750 | 0.08472 |
| 1139.75386 | 0.13333 | 0.04830 | 0.08504 |
| 172.27168 | 0.98889 | 0.90341 | 0.08548 |
| 1510.06024 | 0.10000 | 0.01420 | 0.08580 |
| 775.07472 | 0.21111 | 0.12500 | 0.08611 |
| 314.16979 | 0.70000 | 0.61364 | 0.08636 |
| 816.92381 | 0.18889 | 0.10227 | 0.08662 |
| 870.75766 | 0.17778 | 0.09091 | 0.08687 |
| 917.58052 | 0.16667 | 0.07955 | 0.08712 |
| 402.42727 | 0.53333 | 0.44602 | 0.08731 |
| 995.47162 | 0.15556 | 0.06818 | 0.08737 |
| 406.03019 | 0.52222 | 0.43466 | 0.08756 |
| 1145.84307 | 0.13333 | 0.04545 | 0.08788 |
| 1257.94693 | 0.12222 | 0.03409 | 0.08813 |
| 177.06659 | 0.98889 | 0.90057 | 0.08832 |
| 1531.43955 | 0.10000 | 0.01136 | 0.08864 |
| 779.27298 | 0.21111 | 0.12216 | 0.08895 |
| 314.54784 | 0.70000 | 0.61080 | 0.08920 |
| 827.66088 | 0.18889 | 0.09943 | 0.08946 |
| 881.70985 | 0.17778 | 0.08807 | 0.08971 |
| 403.26512 | 0.53333 | 0.44318 | 0.09015 |
| 1026.60972 | 0.15556 | 0.06534 | 0.09021 |
| 408.32391 | 0.52222 | 0.43182 | 0.09040 |
| 1114.01648 | 0.14444 | 0.05398 | 0.09047 |
| 1157.50010 | 0.13333 | 0.04261 | 0.09072 |
| 1267.90789 | 0.12222 | 0.03125 | 0.09097 |
| 1532.91474 | 0.10000 | 0.00852 | 0.09148 |
| 782.67574 | 0.21111 | 0.11932 | 0.09179 |
| 315.37070 | 0.70000 | 0.60795 | 0.09205 |
| 403.83959 | 0.53333 | 0.44034 | 0.09299 |
| 1037.55080 | 0.15556 | 0.06250 | 0.09306 |
| 412.06190 | 0.52222 | 0.42898 | 0.09324 |
| 1180.01889 | 0.13333 | 0.03977 | 0.09356 |
| 316.89206 | 0.70000 | 0.60511 | 0.09489 |
| 1051.71004 | 0.15556 | 0.05966 | 0.09590 |
| 414.31818 | 0.52222 | 0.42614 | 0.09609 |
| 1198.70418 | 0.13333 | 0.03693 | 0.09640 |
| 769.46002 | 0.22222 | 0.12500 | 0.09722 |
| 317.92016 | 0.70000 | 0.60227 | 0.09773 |
| 397.58762 | 0.54444 | 0.44602 | 0.09842 |
| 339.78908 | 0.65556 | 0.55682 | 0.09874 |
| 1078.33396 | 0.15556 | 0.05682 | 0.09874 |
| 416.30000 | 0.52222 | 0.42330 | 0.09893 |
| 1228.29720 | 0.13333 | 0.03409 | 0.09924 |
| 683.69897 | 0.25556 | 0.15625 | 0.09931 |
| 318.28119 | 0.70000 | 0.59943 | 0.10057 |
| 325.87299 | 0.68889 | 0.58807 | 0.10082 |
| 341.10487 | 0.65556 | 0.55398 | 0.10158 |
| 1102.55598 | 0.15556 | 0.05398 | 0.10158 |
| 419.35901 | 0.52222 | 0.42045 | 0.10177 |
| 701.02155 | 0.25556 | 0.15341 | 0.10215 |
| 437.39178 | 0.50000 | 0.39773 | 0.10227 |
| 725.97493 | 0.24444 | 0.14205 | 0.10240 |
| 749.52526 | 0.23333 | 0.13068 | 0.10265 |
| 319.15134 | 0.70000 | 0.59659 | 0.10341 |
| 327.06636 | 0.68889 | 0.58523 | 0.10366 |
| 338.24026 | 0.66667 | 0.56250 | 0.10417 |
| 341.40339 | 0.65556 | 0.55114 | 0.10442 |
| 423.39554 | 0.52222 | 0.41761 | 0.10461 |
| 430.54575 | 0.51111 | 0.40625 | 0.10486 |
| 709.77778 | 0.25556 | 0.15057 | 0.10499 |
| 439.48613 | 0.50000 | 0.39489 | 0.10511 |
| 729.29161 | 0.24444 | 0.13920 | 0.10524 |
| 757.96567 | 0.23333 | 0.12784 | 0.10549 |
| 320.62005 | 0.70000 | 0.59375 | 0.10625 |
| 327.83070 | 0.68889 | 0.58239 | 0.10650 |
| 390.73932 | 0.55556 | 0.44886 | 0.10669 |
| 338.33196 | 0.66667 | 0.55966 | 0.10701 |
| 341.54625 | 0.65556 | 0.54830 | 0.10726 |
| 425.92410 | 0.52222 | 0.41477 | 0.10745 |
| 668.26638 | 0.26667 | 0.15909 | 0.10758 |
| 433.14242 | 0.51111 | 0.40341 | 0.10770 |
| 716.92185 | 0.25556 | 0.14773 | 0.10783 |
| 440.54071 | 0.50000 | 0.39205 | 0.10795 |
| 732.91516 | 0.24444 | 0.13636 | 0.10808 |
| 765.15981 | 0.23333 | 0.12500 | 0.10833 |
| 321.48516 | 0.70000 | 0.59091 | 0.10909 |
| 328.91240 | 0.68889 | 0.57955 | 0.10934 |
| 392.39966 | 0.55556 | 0.44602 | 0.10953 |
| 614.34611 | 0.30000 | 0.19034 | 0.10966 |
| 338.53349 | 0.66667 | 0.55682 | 0.10985 |
| 342.21978 | 0.65556 | 0.54545 | 0.11010 |
| 427.00060 | 0.52222 | 0.41193 | 0.11029 |
| 670.25149 | 0.26667 | 0.15625 | 0.11042 |
| 434.76909 | 0.51111 | 0.40057 | 0.11054 |
| 721.12572 | 0.25556 | 0.14489 | 0.11067 |
| 441.79764 | 0.50000 | 0.38920 | 0.11080 |
| 736.30506 | 0.24444 | 0.13352 | 0.11092 |
| 463.91927 | 0.47778 | 0.36648 | 0.11130 |
| 384.21951 | 0.57778 | 0.46591 | 0.11187 |
| 323.36403 | 0.70000 | 0.58807 | 0.11193 |
| 329.64946 | 0.68889 | 0.57670 | 0.11218 |
| 618.66056 | 0.30000 | 0.18750 | 0.11250 |
| 343.24103 | 0.65556 | 0.54261 | 0.11294 |
| 428.44041 | 0.52222 | 0.40909 | 0.11313 |
| 435.27159 | 0.51111 | 0.39773 | 0.11338 |
| 723.17507 | 0.25556 | 0.14205 | 0.11351 |
| 443.16288 | 0.50000 | 0.38636 | 0.11364 |
| 742.92113 | 0.24444 | 0.13068 | 0.11376 |
| 466.76203 | 0.47778 | 0.36364 | 0.11414 |
| 381.59037 | 0.58889 | 0.47443 | 0.11446 |
| 483.57171 | 0.45556 | 0.34091 | 0.11465 |
| 385.47239 | 0.57778 | 0.46307 | 0.11471 |
| 330.83726 | 0.68889 | 0.57386 | 0.11503 |
| 337.20574 | 0.67778 | 0.56250 | 0.11528 |
| 621.82684 | 0.30000 | 0.18466 | 0.11534 |
| 343.86713 | 0.65556 | 0.53977 | 0.11578 |
| 659.58931 | 0.27778 | 0.16193 | 0.11585 |
| 429.23978 | 0.52222 | 0.40625 | 0.11597 |
| 444.06796 | 0.50000 | 0.38352 | 0.11648 |
| 468.57705 | 0.47778 | 0.36080 | 0.11698 |
| 382.10968 | 0.58889 | 0.47159 | 0.11730 |
| 484.37866 | 0.45556 | 0.33807 | 0.11749 |
| 386.82829 | 0.57778 | 0.46023 | 0.11755 |
| 389.58889 | 0.56667 | 0.44886 | 0.11780 |
| 332.41832 | 0.68889 | 0.57102 | 0.11787 |
| 625.20528 | 0.30000 | 0.18182 | 0.11818 |
| 344.09476 | 0.65556 | 0.53693 | 0.11862 |
| 664.25220 | 0.27778 | 0.15909 | 0.11869 |
| 444.61131 | 0.50000 | 0.38068 | 0.11932 |
| 469.39763 | 0.47778 | 0.35795 | 0.11982 |
| 383.19846 | 0.58889 | 0.46875 | 0.12014 |
| 485.20547 | 0.45556 | 0.33523 | 0.12033 |
| 387.82335 | 0.57778 | 0.45739 | 0.12039 |
| 333.10145 | 0.68889 | 0.56818 | 0.12071 |
| 608.75017 | 0.31111 | 0.19034 | 0.12077 |
| 625.95342 | 0.30000 | 0.17898 | 0.12102 |
| 642.03704 | 0.28889 | 0.16761 | 0.12128 |
| 344.34522 | 0.65556 | 0.53409 | 0.12146 |
| 445.28335 | 0.50000 | 0.37784 | 0.12216 |
| 460.73465 | 0.48889 | 0.36648 | 0.12241 |
| 469.87876 | 0.47778 | 0.35511 | 0.12266 |
| 380.47125 | 0.60000 | 0.47727 | 0.12273 |
| 480.84492 | 0.46667 | 0.34375 | 0.12292 |
| 383.97403 | 0.58889 | 0.46591 | 0.12298 |
| 485.61927 | 0.45556 | 0.33239 | 0.12317 |
| 388.60413 | 0.57778 | 0.45455 | 0.12323 |
| 334.54113 | 0.68889 | 0.56534 | 0.12355 |
| 629.30652 | 0.30000 | 0.17614 | 0.12386 |
| 644.54651 | 0.28889 | 0.16477 | 0.12412 |
| 344.89423 | 0.65556 | 0.53125 | 0.12431 |
| 448.23459 | 0.50000 | 0.37500 | 0.12500 |
| 471.92949 | 0.47778 | 0.35227 | 0.12551 |
| 381.16364 | 0.60000 | 0.47443 | 0.12557 |
| 482.32567 | 0.46667 | 0.34091 | 0.12576 |
| 486.84211 | 0.45556 | 0.32955 | 0.12601 |
| 388.83619 | 0.57778 | 0.45170 | 0.12607 |
| 335.98929 | 0.68889 | 0.56250 | 0.12639 |
| 633.13197 | 0.30000 | 0.17330 | 0.12670 |
| 651.72407 | 0.28889 | 0.16193 | 0.12696 |
| 346.18858 | 0.65556 | 0.52841 | 0.12715 |
| 452.83427 | 0.50000 | 0.37216 | 0.12784 |
| 474.94540 | 0.47778 | 0.34943 | 0.12835 |
| 597.76409 | 0.33333 | 0.20455 | 0.12879 |
| 488.91832 | 0.45556 | 0.32670 | 0.12885 |
| 389.06889 | 0.57778 | 0.44886 | 0.12891 |
| 605.99591 | 0.32222 | 0.19318 | 0.12904 |
| 634.09023 | 0.30000 | 0.17045 | 0.12955 |
| 348.61963 | 0.65556 | 0.52557 | 0.12999 |
| 456.57354 | 0.50000 | 0.36932 | 0.13068 |
| 477.10691 | 0.47778 | 0.34659 | 0.13119 |
| 598.19092 | 0.33333 | 0.20170 | 0.13163 |
| 490.34408 | 0.45556 | 0.32386 | 0.13169 |
| 606.85611 | 0.32222 | 0.19034 | 0.13188 |
| 637.34438 | 0.30000 | 0.16761 | 0.13239 |
| 350.08948 | 0.65556 | 0.52273 | 0.13283 |
| 458.84926 | 0.50000 | 0.36648 | 0.13352 |
| 378.26590 | 0.61111 | 0.47727 | 0.13384 |
| 479.29335 | 0.47778 | 0.34375 | 0.13403 |
| 587.19384 | 0.34444 | 0.21023 | 0.13422 |
| 601.22683 | 0.33333 | 0.19886 | 0.13447 |
| 491.07289 | 0.45556 | 0.32102 | 0.13453 |
| 351.87102 | 0.65556 | 0.51989 | 0.13567 |
| 591.41064 | 0.34444 | 0.20739 | 0.13706 |
| 604.71028 | 0.33333 | 0.19602 | 0.13731 |
| 496.14287 | 0.45556 | 0.31818 | 0.13737 |
| 353.65696 | 0.65556 | 0.51705 | 0.13851 |
| 373.21117 | 0.62222 | 0.48295 | 0.13927 |
| 584.46126 | 0.35556 | 0.21591 | 0.13965 |
| 596.57778 | 0.34444 | 0.20455 | 0.13990 |
| 605.44532 | 0.33333 | 0.19318 | 0.14015 |
| 502.30275 | 0.45556 | 0.31534 | 0.14021 |
| 353.96764 | 0.65556 | 0.51420 | 0.14135 |
| 373.94216 | 0.62222 | 0.48011 | 0.14211 |
| 585.60455 | 0.35556 | 0.21307 | 0.14249 |
| 504.39701 | 0.45556 | 0.31250 | 0.14306 |
| 539.35467 | 0.41111 | 0.26705 | 0.14407 |
| 354.30337 | 0.65556 | 0.51136 | 0.14419 |
| 375.41566 | 0.62222 | 0.47727 | 0.14495 |
| 586.46898 | 0.35556 | 0.21023 | 0.14533 |
| 505.09265 | 0.45556 | 0.30966 | 0.14590 |
| 513.96131 | 0.44444 | 0.29830 | 0.14615 |
| 541.93481 | 0.41111 | 0.26420 | 0.14691 |
| 356.19631 | 0.65556 | 0.50852 | 0.14703 |
| 370.47425 | 0.63333 | 0.48580 | 0.14754 |
| 505.79901 | 0.45556 | 0.30682 | 0.14874 |
| 516.86984 | 0.44444 | 0.29545 | 0.14899 |
| 544.29249 | 0.41111 | 0.26136 | 0.14975 |
| 358.32109 | 0.65556 | 0.50568 | 0.14987 |
| 372.18445 | 0.63333 | 0.48295 | 0.15038 |
| 580.81039 | 0.36667 | 0.21591 | 0.15076 |
| 508.55045 | 0.45556 | 0.30398 | 0.15158 |
| 519.44065 | 0.44444 | 0.29261 | 0.15183 |
| 544.74744 | 0.41111 | 0.25852 | 0.15259 |
| 358.73531 | 0.65556 | 0.50284 | 0.15271 |
| 562.55665 | 0.38889 | 0.23580 | 0.15309 |
| 511.79056 | 0.45556 | 0.30114 | 0.15442 |
| 520.34620 | 0.44444 | 0.28977 | 0.15467 |
| 526.92190 | 0.43333 | 0.27841 | 0.15492 |
| 537.97545 | 0.42222 | 0.26705 | 0.15518 |
| 545.41786 | 0.41111 | 0.25568 | 0.15543 |
| 358.95055 | 0.65556 | 0.50000 | 0.15556 |
| 367.35902 | 0.64444 | 0.48864 | 0.15581 |
| 563.08637 | 0.38889 | 0.23295 | 0.15593 |
| 513.18433 | 0.45556 | 0.29830 | 0.15726 |
| 522.12927 | 0.44444 | 0.28693 | 0.15751 |
| 529.92490 | 0.43333 | 0.27557 | 0.15777 |
| 547.49404 | 0.41111 | 0.25284 | 0.15827 |
| 360.29204 | 0.65556 | 0.49716 | 0.15840 |
| 557.91905 | 0.40000 | 0.24148 | 0.15852 |
| 368.85656 | 0.64444 | 0.48580 | 0.15865 |
| 564.15266 | 0.38889 | 0.23011 | 0.15878 |
| 573.09312 | 0.37778 | 0.21875 | 0.15903 |
| 523.19515 | 0.44444 | 0.28409 | 0.16035 |
| 531.11783 | 0.43333 | 0.27273 | 0.16061 |
| 549.65888 | 0.41111 | 0.25000 | 0.16111 |
| 361.87548 | 0.65556 | 0.49432 | 0.16124 |
| 559.94871 | 0.40000 | 0.23864 | 0.16136 |
| 565.55329 | 0.38889 | 0.22727 | 0.16162 |
| 575.78643 | 0.37778 | 0.21591 | 0.16187 |
| 523.45797 | 0.44444 | 0.28125 | 0.16319 |
| 534.12633 | 0.43333 | 0.26989 | 0.16345 |
| 550.79898 | 0.41111 | 0.24716 | 0.16395 |
| 363.40691 | 0.65556 | 0.49148 | 0.16408 |
| 561.62666 | 0.40000 | 0.23580 | 0.16420 |
| 565.98525 | 0.38889 | 0.22443 | 0.16446 |
| 523.93225 | 0.44444 | 0.27841 | 0.16604 |
| 536.60627 | 0.43333 | 0.26705 | 0.16629 |
| 553.05149 | 0.41111 | 0.24432 | 0.16679 |
| 365.53453 | 0.65556 | 0.48864 | 0.16692 |
| 567.95570 | 0.38889 | 0.22159 | 0.16730 |
| 555.82420 | 0.41111 | 0.24148 | 0.16963 |
| 571.28545 | 0.38889 | 0.21875 | 0.17014 |
